# URBAN ENGAGEMENT STRATEGIES FOR ALASKA NATIVE ELDER PARTICIPATION IN RESEARCH

**DOI:** 10.1093/geroni/igac059.1514

**Published:** 2022-12-20

**Authors:** Steffi Kim

**Affiliations:** University of Minnesota, Minneapolis, Minnesota, United States

## Abstract

Given the current threats to health for older Indigenous people, it is important to understand the health needs and perceptions of older Indigenous people who play a pivotal role in the health and wellbeing of their communities. The underrepresentation of Indigenous voices has led to research, health promotion efforts, and community engagement that has largely been unsuccessful in facilitating culturally grounded and effective health promotion. Engaging Indigenous Elders in research has often been a challenge for researchers – Indigenous and non-Indigenous. Reaching Indigenous Elders within the urban environment can even be more challenging at times based on dispersed communities due to challenging historical contexts, experiences of racism, and mistrust. This presentation is based on the employed engagement strategies of urban-based Alaska Native Elders. Developing policies and programs to promote healthy aging in Indigenous communities requires the voices of those living within their communities.

